# Unprecedented heat wave in December 2015 and potential for winter glacier ablation in the eastern Alps

**DOI:** 10.1038/s41598-017-07415-1

**Published:** 2017-08-02

**Authors:** Renato R. Colucci, Filippo Giorgi, Csaba Torma

**Affiliations:** 1 Department of Earth System Sciences and Environmental Technology, ISMAR-CNR Trieste, I-34149 Italy; 2 Earth System Physics Section, The Abdus Salam International Centre for Theoretical Physics, Trieste, I-34151 Italy; 3 Department of Meteorology, Eötvös Loránd University and HAS Post-Doctoral Research Program, Budapest, Hungary

## Abstract

We document the occurrence in December 2015 of unprecedented high monthly mean temperatures in the observational record of mountain sites in the eastern Alps. For the first time in the last 150 years mean December temperature exceeded 0 °C at elevations between 2100 and 2500 m, with December mean anomalies exceeding 6.5 °C with respect to the 1971–2000 mean. Along with the absence of snow cover, such temperatures might have lead to unprecedented winter ablation of glaciers in this elevation range. Smaller temperature anomalies occurred in surrounding low elevation sites, highlighting the key role of topography in this event. Specifically, strong inversions associated with the very stable synoptic conditions during the month amplified the anomalies at the high elevations of the mountain glacier sites. We analyze the processes underlying this exceptional event and place this anomaly within the context of future warming scenarios over the region.

## Introduction

Recent decades have been characterized by the occurrence of heat waves and warm periods of exceptional magnitude, such as the summer of 2003 in Europe^1^, the 2010 heat wave in Russia^2^, or the summer of 2013 in Australia^3^. Since global warming is expected to yield an increase in the severity of heat waves^4, 5^, it has been hypothesized that the ongoing warming trend could influence the occurrence of these events^1, 6^. Indeed, the increase in heat wave intensity and frequency is one of the most prominent and robust signatures of global warming projections^5–7^. While the attribution of individual heat wave events to anthropogenic greenhouse gas (GHG) forcing remains difficult, it can be expected that the probability of occurrence of increasingly severe warm episodes of unprecedented magnitude will increase with global warming^8, 9^.

This issue is particularly relevant for mountain environments, since different studies have shown that topography can locally amplify the surface warming^10–12^. This amplification can occur in response to several processes: i) the effect of the snow-albedo feedback mechanism associated with a decrease in snow cover in warmer conditions, which is especially relevant in the spring season and at intermediate elevations^10–12^; ii) the effect of changes in vertical temperature lapse rate, a process effective in all seasons but with different vertical characteristics^11^; iii) changes in cloud cover and water vapor profiles^12^; iv) changes in aerosol distributions, such as dust and black carbon^12^. In addition, while summer heat waves have drawn much attention because of their impacts on human health and ecosystem resiliency^13, 14^, wintertime warming and heat extremes can also have important consequences^15^. For example changes in winter temperature and precipitation regimes in mountain environments can affect glacier accumulation/ablation, water resources, ecosystem dynamics and biodiversity, forestry, natural hazards, tourism and recreational activities^15^.

In particular, mountain glaciers are considered to be sensitive indicators of climate variability^16^. They react in a relatively simple way to changes in climate, as their mass balance mainly depends on variables such as air temperature, precipitation, humidity and wind speed^16, 17^. The seasonal glacier mass balances depend on the climate regime such as the timing and length of the warm and cold seasons, and the partitioning of precipitation between rain and snow^18^. In this regard, European Alpine glaciers have well-defined winter accumulation and summer ablation seasons due to a pronounced seasonal temperature variation^19^. Therefore, the mass of Alpine glaciers is at a minimum towards the end of the summer ablation season, just prior to the first snow events of the cold season. For some low-altitude glacier tongues and ice patches, minor ablation may occasionally occur in the winter during the passage of warm fronts. However, for such glaciers lying at temperatures not far from the melting point, large warm anomalies have the potential of affecting the mass balance also during the winter.

Globally, the year 2015 has been the second warmest (after 2016) on record in the last 150 years^20^, continuing a long term warming trend whose main contribution has been attributed with a high degree of confidence (95–100%) to anthropogenic GHG-induced forcing^21^. It is thus likely that this record setting year might have produced extreme regional manifestations that could significantly affect local hydro-climatic budgets. Specifically, the late fall/early winter season of 2015 was characterized by exceptionally warm, dry and stable synoptic conditions throughout central-eastern Europe^22^, with the potential of strong anomalies over the eastern Alpine region, particularly at mid to high elevations, due to the occurrence of strong thermal inversions. We thus analyzed a set of high and low elevation climate records^23, 24^ for the early winter (December) over the eastern Alps dating back to the mid 19th century to assess the characteristics of December 2015 within the context of these long term records and of ongoing climatic trends over the region.

## Results and Discussion

### Unprecedented temperatures in December 2015 at high elevation sites of the Eastern Alps

**Figure 1:**
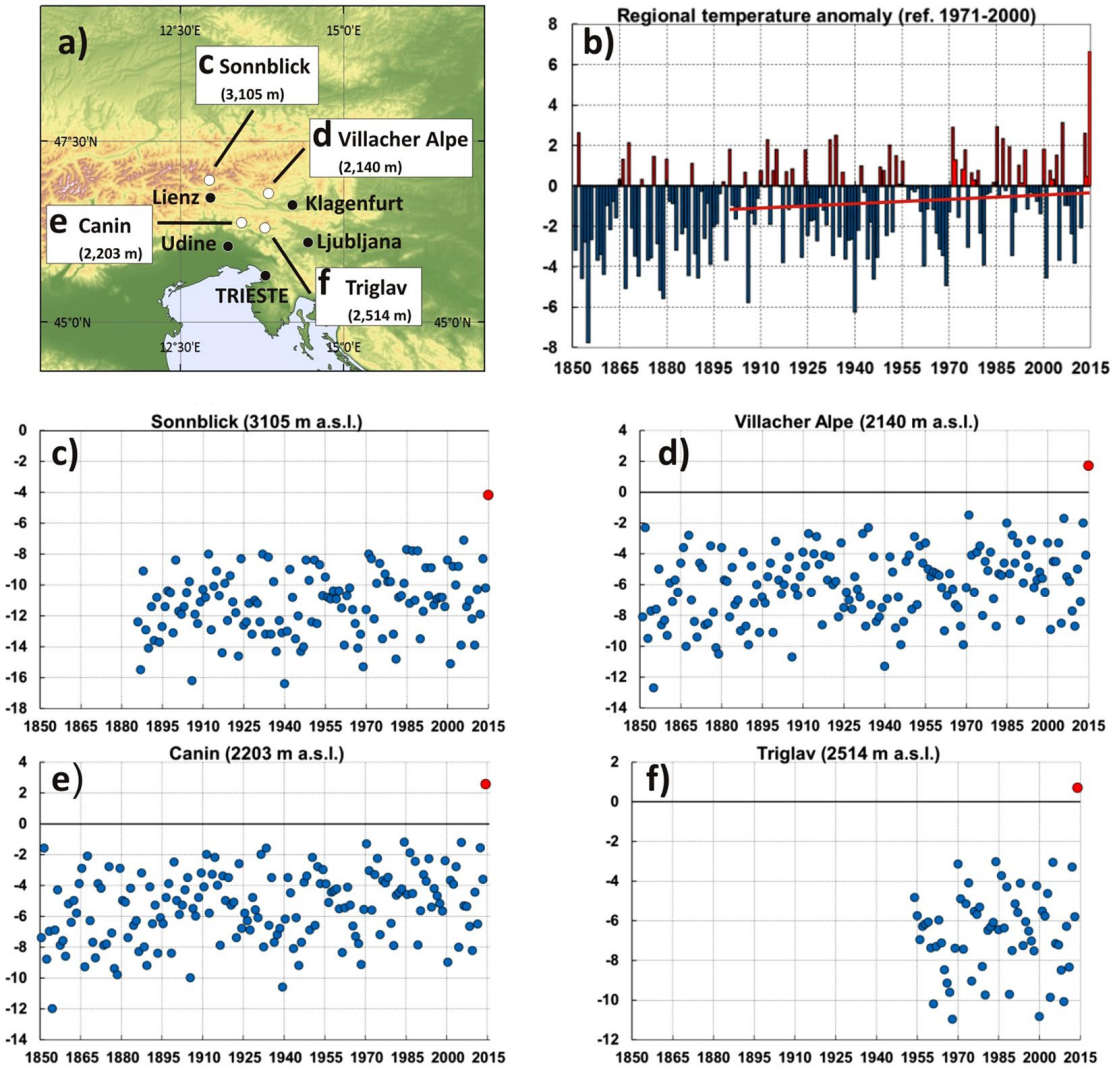
Observed mean December temperature at the four high elevation sites shown in the top left panel (a) (**c**, Sonnblick; **d**, Villacher Alpe; **e**, Canin; **f** Triglav). This map has been created with ArcMap version 10.3 and is based on open access digital elevation information from the Shuttle Radar Topography Mission (http://srtm.csi.cgiar.org), further edited by using the CorelDRAW graphic suite, release ×3 (http://www.corel.com). The temperature anomaly (compared to the 1971–2000 mean) averaged over the four sites is shown in the top right panel (b). Units are °C.

**Figure 2:**
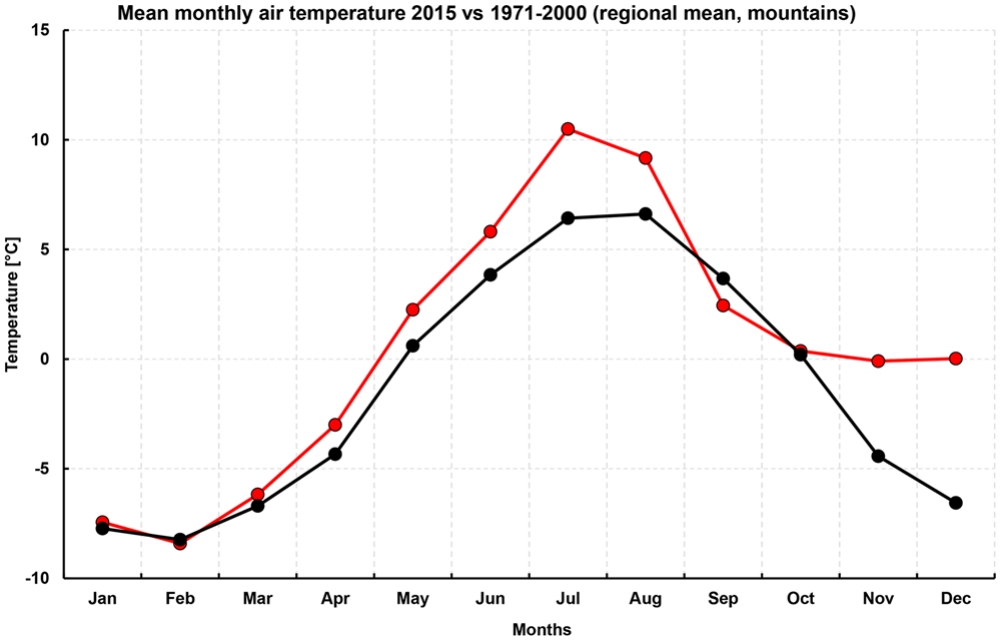
Annual cycle of monthly temperature averaged over the four high elevation sites of Fig. 1 in 2015 (red curve) and for the 1971–2000 mean (black curve).

Figure 1 shows the December mean temperature record at four high elevation sites in the eastern Alps: Canin (2203 m), Triglav (2514 m), Villacher Alpe (2140 m), Sonnblick (3150 m). The records start in 1851 for Canin and Villacher Alpe, 1886 for Sonnblick and 1954 for Triglav. Also shown are the December mean temperature anomalies (compared to the reference period 1971–2000) averaged over the four sites (or over those of the four sites with available data for a given year), along with the linear trend from the beginning of the 20^th^ century to 2015 (the period most affected by anthropogenic greenhouse gas induced warming)^21^. Looking first at the cross-site average (Fig. 1b), which is representative of regional high elevation conditions, a positive temperature anomaly of exceptional magnitude, about 6.5 °C, is observed in December 2015. The December 2015 anomaly is 3.72 standard deviations from the 1971–2000 mean, corresponding to a probability of occurrence of about 10^−4^ assuming a Gaussian distribution of seasonal temperatures. Also evident is a warming trend throughout the 20th century December record, with an increasing number of positive anomalies after the middle of the century. The trend in December temperatures averaged over the four sites is about 0.0107 °C yr^−1^ since 1900, implying a mean warming of about 1.24 °C throughout the 116 yr period. An analysis of the records at individual sites reveals that, superimposed to the 20th century warming trend, for the first time values of December mean temperatures above the 0 °C are found in 2015 at elevations between 2100 m and 2500 m, while for the first time the mean temperature is close to −4 °C at the higher elevation site of Sonnblick. Figure 2 compares the 2015 annual cycle of monthly temperature with the corresponding long term (1971–2000) mean annual cycle, averaged over the four sites. It shows that, although substantial positive anomalies also occurred in July and November 2015, the December 2015 anomaly is by far the most pronounced. Essentially, the 2015 monthly mean temperature at the high elevation sites remained about the same from October through December.

By comparison, Supplementary Fig. S1 shows that neighboring low elevation sites in the region (Udine, Ljubljana, Klagenfurt, and Lienz) exhibited December 2015 temperatures in line with the long term warming trends, with 2015 being not as extreme as at high elevations, and in some cases (e.g. Klagenfurt) even close to the average values. This indicates that temperature inversions and elevation played a key role in determining the unprecedented surface temperature anomalies of December 2015 at the high elevation sites.

**Figure 3:**
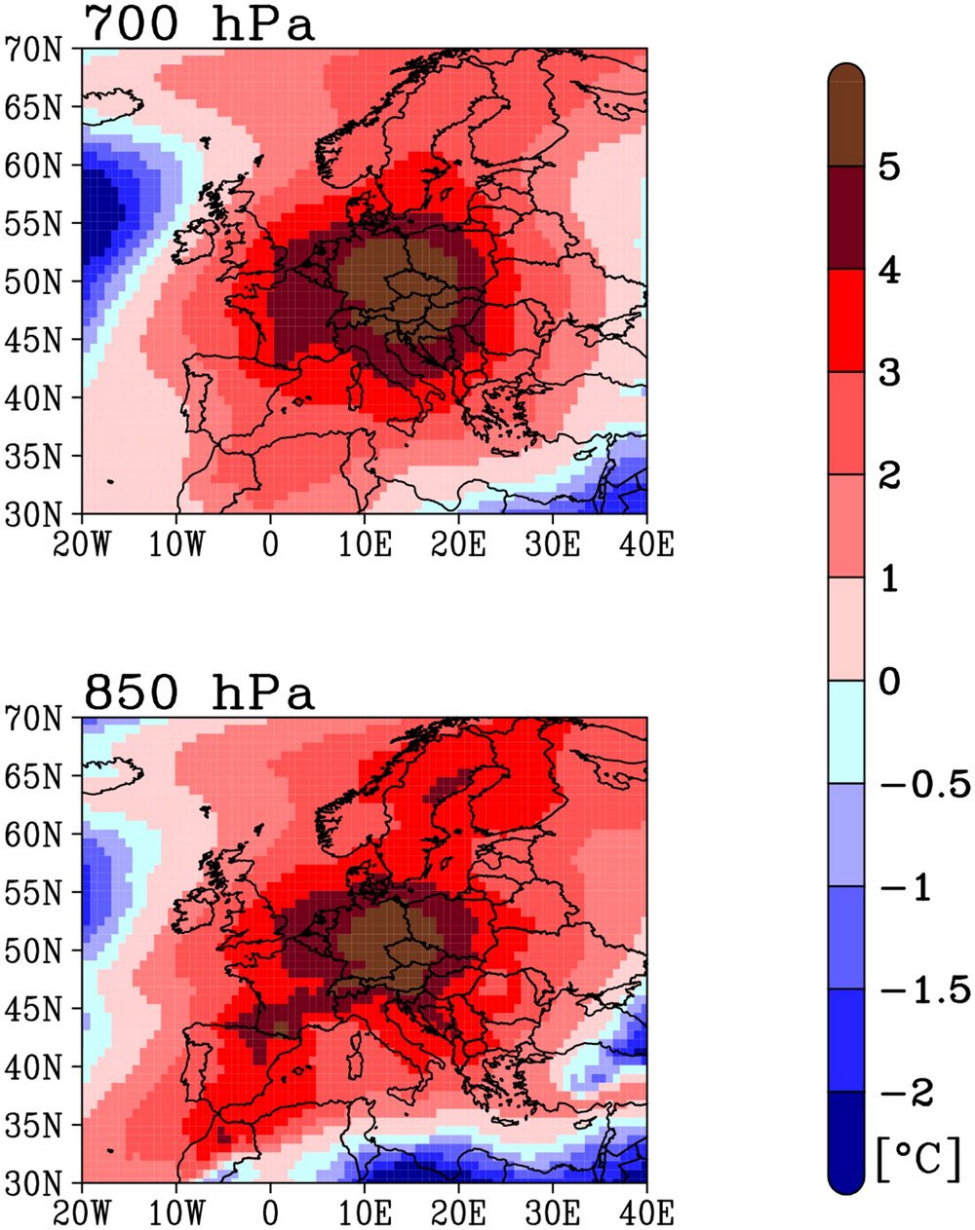
December temperature anomalies at 700 hPa and 850 hPa from the ERA-Interim reanalysis (http://apps.ecmwf.int/datasets/) for 2015, compared to the reference period 1981–2010. The map was created with the software Grads (http://cola.gmu.edu/grads/).

What were the synoptic conditions underlying this event? Fig. 3 presents the temperature anomalies at 850 hPa and 700 hPa for December 2015 (compared to the 1981–2010 December mean, since ERA-Interim is only available starting 1979) from the ERA-Interim reanalysis^20^. It shows that the warm anomaly occurred at the regional scale, covering essentially the entire European continent, with a sharp maximum of up to 5 °C centered over central-eastern Europe.

**Figure 4:**
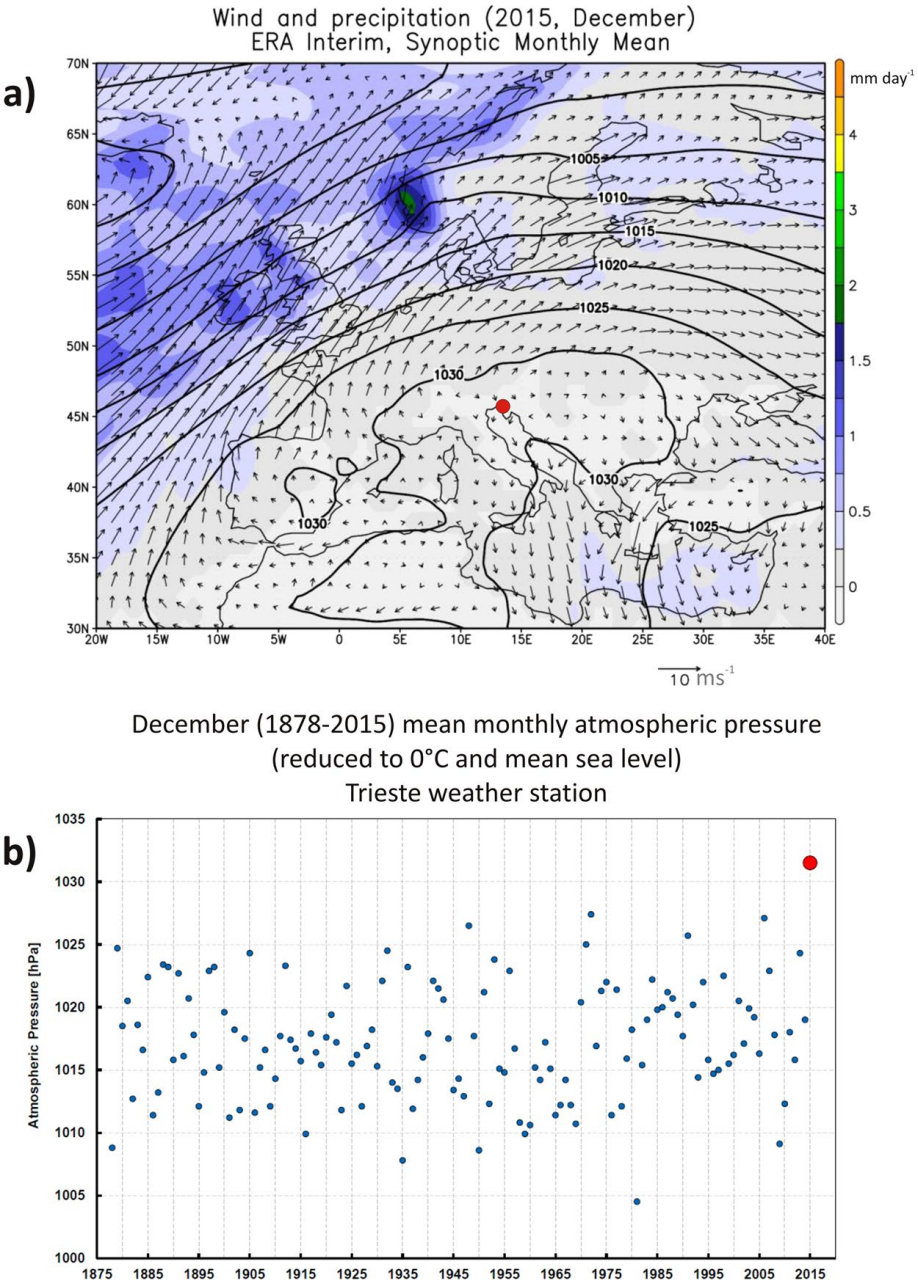
Top panel (a): December 2015 sea level pressure (hPa, contour lines), precipitation (mm/day, colors) and surface wind (m/s, arrows) from the ERA-Interim reanalysis (http://apps.ecmwf.int/datasets/). The map has been created with the software Grads (http://cola.gmu.edu/grads/). Bottom panel (b): time series of December sea level pressure (hPa) at Trieste (red dot in panel a), located in the northeastern Adriatic coasts of Italy.

Figure 4a shows the sea level pressure (SLP) along with the near-surface (10 meters) mean wind and precipitation for December 2015 from the ERA-Interim reanalysis^22^. A deep anticyclonic blocking high pressure area persisted over the European continent during the month, with center over southeastern Europe and a pronounced ridge aloft. Figure 4b also reports the long term SLP record for the coastal site of Trieste dating back to 1878. In agreement with the regional scale analysis, a strong positive anomaly is found in December 2015, with an unprecedented mean SLP value above 1030 hPa. This SLP anomaly over the southeastern European region lead to stable conditions and downwelling motion over the area, which in turn favored the inception of pronounced inversion layers yielding the vertical gradient in temperature anomaly found between low elevation and high mountain sites. Examples of observed temperature inversions from radiosonde data during December 2015 at Rivolto (close to Udine) and Zagreb can be found at the web site http://weather.uwyo.edu/upperair/sounding.html.

To complement the information of Fig. 4, Supplementary Fig. S2 shows the long term December precipitation records for the four low elevation sites in the eastern Alpine region as well as the Trieste site. At all sites, essentially no or negligible precipitation occurred in December 2015, a further evidence of the strongly stable weather conditions which occurred during this month. We also note that these near zero precipitation amounts ( < 1 mm), although not unprecedented, are found only in less than 3% of the years within the long term records at the selected stations.

### The extreme warm temperatures of December 2015 might have caused winter glacier ablation

One of the consequences of the extreme warm anomalies found in December 2015 over the eastern Alps is the possibility of winter glacier ablation in the area. As mentioned, winter ablation does occur occasionally in the Alps, being generally linked to relatively warm rain water falling on glacier surfaces during fast passages of warm fronts. This takes place especially in environments where the maritime influence is stronger^25^, such as the southern side of the alpine chain^26, 27^. However, in general, rain is only a minor component of the energy balance of glaciers, and melting phenomena occur only if the snow or ice is already at the melting point, which is normally not the case in December over the Alps.

As also shown in Fig. 4, in the fall/early winter 2015 only very little precipitation fell over the eastern Alpine region, and indeed only a single moderate snow event occurred in November. Sustained high temperatures and incoming solar radiation almost completely removed snow from the ground by the end of December (Supplementary Fig. S3 showing the Canin ice patches at 2200 m in October and December, 2015). Parts of glaciers and ice patches, especially those at the lower elevations, had ice exposed, and these conditions would be able to cause ablation under the above-freezing temperatures found in December 2015.

In order to provide an estimate of this effect, we calculated the potential of ice melting during December 2015 at the Canin, Triglav and Sonnblick sites, where ice patches and small glaciers are present^26, 28–30^, by using a degree-day factor (DDF) approach for melting snow and ice. We used a DDF for melting snow of 4.1 mm day^−1^ °C^−1^ water equivalent (w.e.) and a DDF for melting glacier ice of 7.9 mm day^−1^ °C, which are widely adopted to estimate ice-snow ablation at the Equilibrium Line Altitude (ELA) of glaciers in different regions of the world^28^. Note that in previous studies the DDF was calculated for the ablation season (mostly summer) and, since the DDF implicitly integrates radiation information, it may be lower for the winter. Therefore our estimates should be considered as upper values.

Applying these DDFs to the December 2015 daily temperature time series at the three sites leads to ablation estimates of 342 mm w.e. and 667 mm w.e. at Canin (2200 m) respectively for melting snow and ice, 182 mm w.e. (snow) and 355 mm w.e. (ice) at Triglav (2500 m), and 8 mm w.e. (snow) and 16 mm w.e. (ice) at Sonnblick (2900 m). Although these estimates cannot be validated against observations, these simple calculations suggest that significant glacier ablation might have occurred during December 2015 at elevations in the range of 2000–2500 m.

We can also estimate the contribution of global warming to the potential ablation at the three sites in December 2015 by calculating the mean December temperature change since the beginning of the 20th century. This can be done by extrapolating the linear trend of 0.0107 °C yr^−1^ for a period of 116 years (from 1900 to 2015), which gives a contribution of 1.24 °C. Removing this contribution from the December 2015 temperatures yields potential ablation values of 218 mm w.e. (snow) and 426 mm w.e. (ice) at Canin, 103 mm w.e. and 201 mm w.e. at Triglav and 2 and 3 mm w.e. at Sonnblick, suggesting that global warming during the 20^th^ and early 21^st^ centuries roughly increased by 44% the potential for ablation by the December 2015 anomaly at the sites considered.

### The 2015 December temperature anomaly within the context of climate projections over the region

Although it is clearly not possible to attribute the specific December 2015 anomaly to global warming, it is interesting to place this anomaly within the context of possible future climate projections for the region. Indeed, different generations of global and regional climate model projections indicate a strong warming over the European Alps, of up to several °C by the end of the 21st century under the high end scenarios of greenhouse gas (GHG) concentration increase^20, 31, 32^.

**Figure 5:**
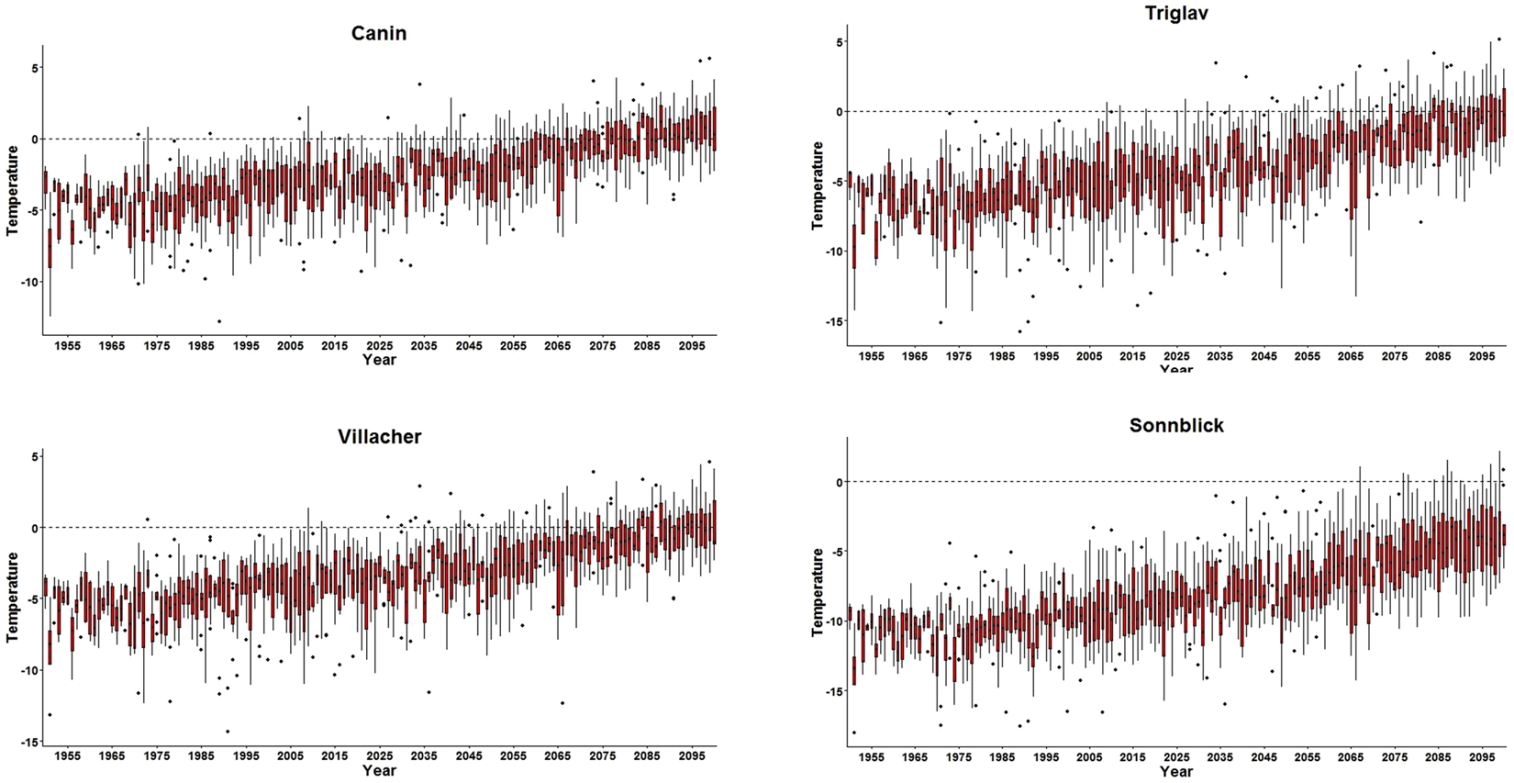
Historical and future December mean temperature (1950–2100, RCP8.5) at the grid points closest to the locations of the four mountain sites of Fig. 1, as simulated by the ensemble of RCMs in Supplementary Table 1, and after a topographical correction is applied (see text). The bar and whiskers plots show the inter-model median, 25th and 75th percentiles and maximum and minimum values in the ensemble. If the distance of a given value from the median is greater than 1.5 times the inter-quantile range, the corresponding value (considered as an outlier) is indicated with a dot.

Figure 5 shows historical and future December mean temperature (1950–2100) at the grid points closest to the locations of the four mountain sites of Fig. 1, as simulated by an ensemble of 10 RCM projections listed in Supplementary Table 1 and completed under the EURO-CORDEX^33^ and Med-CORDEX^34^ initiatives. The RCMs are run at a relatively high horizontal nominal resolution (grid spacing of ~ 12 km) for the high end RCP8.5 GHG scenario^35^. A topographic correction of 6.4 °C/km is applied to the model data to account for differences in elevation between the grid points and the corresponding actual observing sites. Figure 5 reports the median and inter-quantile ranges of temperature across model projections, showing that, even under this extreme warming scenario, December mean temperatures above 0 °C are projected to occur at the Canin, Triglav, and Villacher sites only during the second half of the 21st century. In fact, the December ensemble mean warming anomalies are of the order of 5 °C or more only in the late 21^st^ century.

It is beyond the purpose of this paper to present a detailed analysis of the model projections. We only point out that Fig. 5 suggests that the thermal anomaly found during December 2015 at the mountain sites considered here appears to be characteristic of late 21^st^ century temperature conditions under strong GHG forcing. Given the role played by temperature inversions in amplifying the surface warming at high elevations, a fine scale representation of topography is necessary to capture this effect. As we have already noted, previous analyses of RCM projections have shown that changes in vertical lapse rates, and specifically a mean decrease of the lapse rate associated with more stable conditions, tend to produce increased warming at higher elevations in the winter^11^, although this mean effect is obviously smaller than during the extreme conditions found in December 2015. As the resolution of regional climate models approaches convection-permitting scales (a few km) it will be interesting to examine whether events such as the December 2015 winter will become more frequent and/or intense in the future.

### Final considerations

In this paper we have documented the occurrence of exceptionally warm conditions during December 2015 at high elevations of the eastern Alps. These conditions are unprecedented in the record of the last 150 years, leading to mean December temperatures above 0 °C in the 2100–2500 m elevation range. Because of the almost complete absence of snow fall and the very scarce presence of snow even over accumulation areas of local glaciers in that altitude range, this might have led to unprecedented wintertime glacier ablation, although direct observations to verify this conclusion are not available. Even though the area includes only a few relatively large Alpine valley glaciers flowing at elevations of 2000–2300 m, along with avalanche-fed ice patches and small glaciers which are a specific characteristic of this region, the potential for significant winter glacier ablation still makes the heat wave of December 2015 a remarkable event.

An important aspect of our finding is that this exceptional warm anomaly was found only at high mountain elevations due to thermal inversions associated with extremely stable conditions during the month. Thus, the topography of the eastern Alps was a key element in determining the surface temperature anomaly. High insolation associated with essentially cloud-free conditions in December 2015 likely enhanced to some extent the warm anomaly, while the lower albedo associated with reduced snow cover probably had a minor role, since some of the reporting sites (e.g. Canin) are totally in the shade during December and since solar radiation is low in this season.

This event places itself among the list of recent extreme climate episodes which appear to increasingly occur in line with the ongoing warming under increased GHG forcing, although we cannot attribute it directly to the GHG-induced warming. The exceptional nature of this warm episode, considered within the context of the expected future warming trends, further indicates that global warming may pose increasingly severe threats to mountain environments.

## Electronic supplementary material

Supplementary information
