# Supplementary material for: Unprecedented heat wave in December 2015 and potential for winter glacier ablation in the eastern Alps
Source: Sci Rep. 2017 Aug 2;7:7090. doi: 10.1038/s41598-017-07415-1 (PMC5541132; doi:10.1038/s41598-017-07415-1)
Supplement: Supplementary file 1 — Supplementary information [file 41598_2017_7415_MOESM1_ESM.pdf]

Unprecedented heat wave in December 2015 and potential for winter glacier ablation in the eastern Alps

Renato R. Colucci[1, *], Filippo Giorgi[2], Csaba Torma[2, 3]

Supplementary information

Supplementary Figure S1.

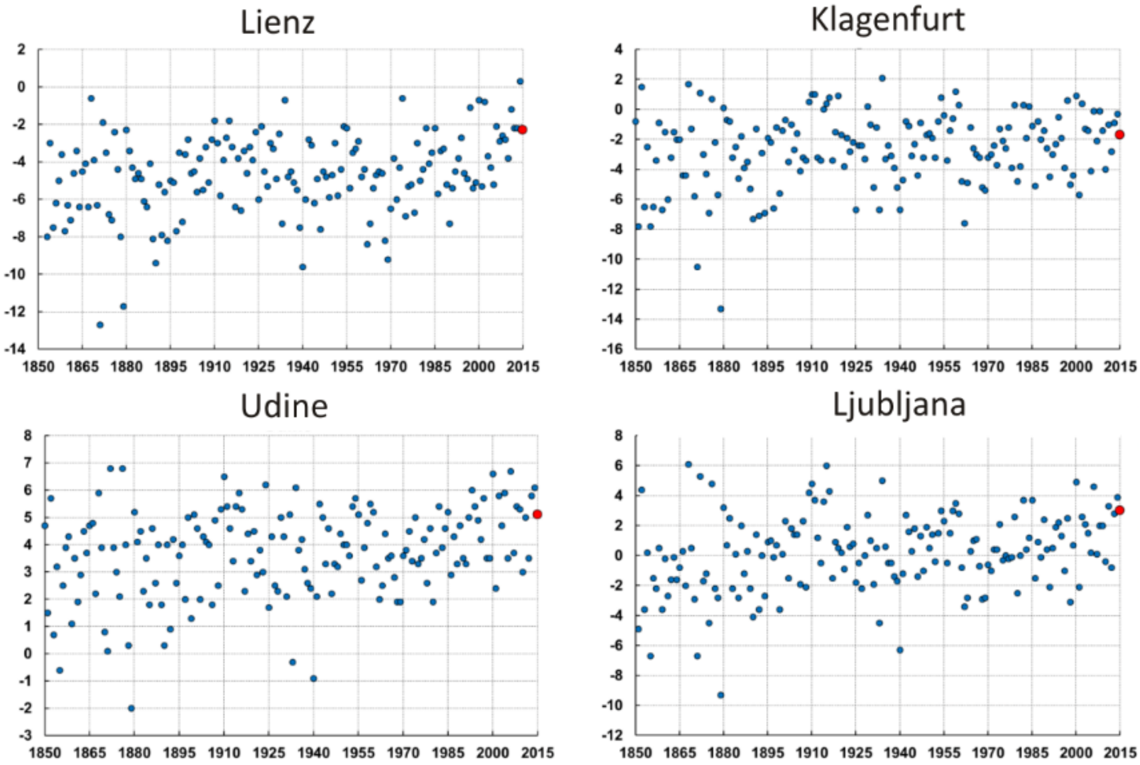

Supplementary Figure S1. December mean temperature record at four low elevation sites close to the mountain sites of Figure 1: Lienz; Klagenfurt; Udine; Ljubljana. Units are ^{o}C. The map has been created with ArcMap version 10.3 and is based on open access digital elevation information from the Shuttle Radar Topography Mission (http://srtm.csi.cgiar.org), further edited by using the CorelDRAW graphic suite, release X3 (http://www.corel.com).

Supplementary Figure S2.

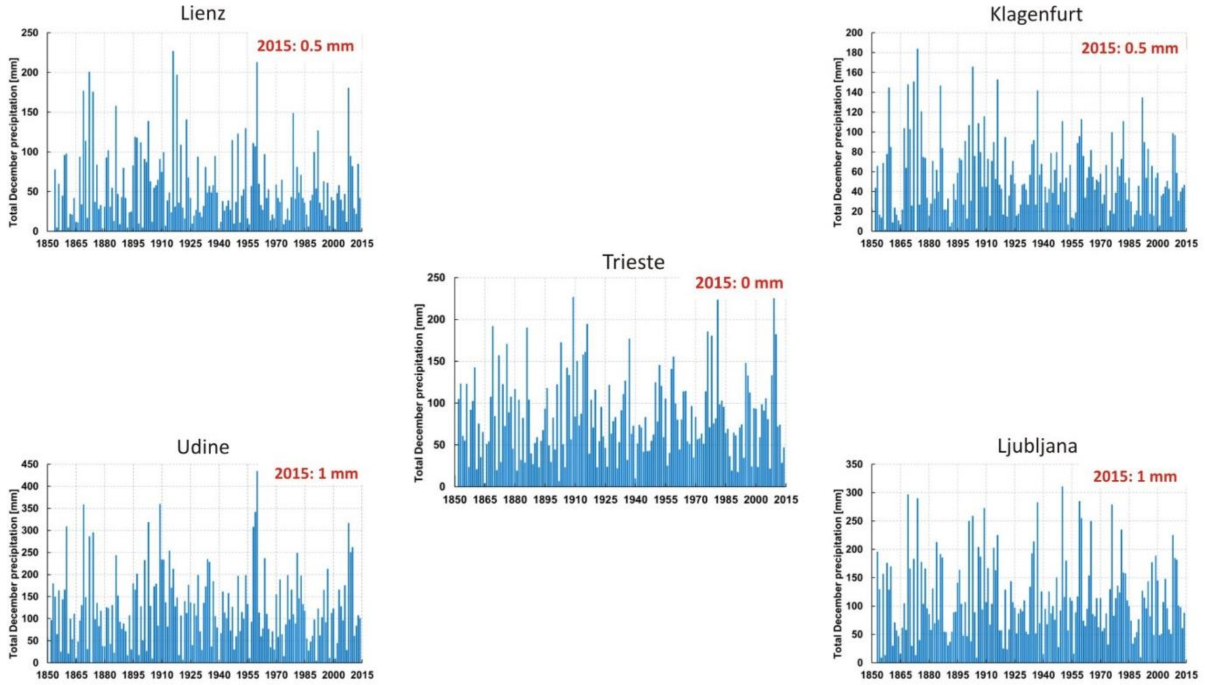

Supplementary Figure S2. December mean precipitation record at five low elevation sites close to the mountain sites of Figure 1: Lienz; Klagenfurt; Udine; Ljubljana; Trieste. Units are mm/day. The map has been created with ArcMap version 10.3 and is based on open access digital elevation information from the Shuttle Radar Topography Mission (http://srtm.csi.cgiar.org), further edited by using the CorelDRAW graphic suite, release X3 (http://www.corel.com).

Supplementary Figure S3.

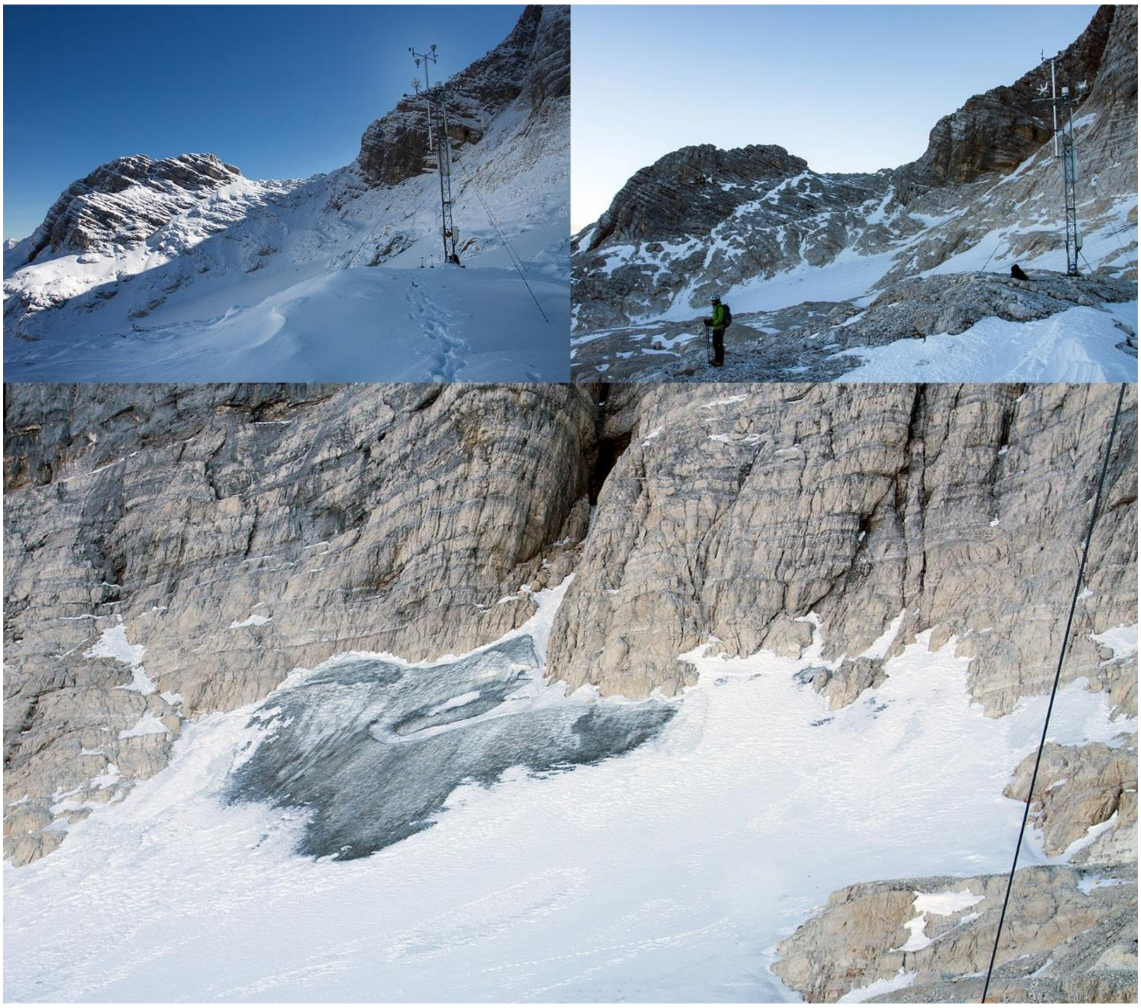

Supplementary Figure S3. Photographs of the Canin plateau at 2200 m a.s.l. taken by Renato R. Colucci on 20th October (top left) and 30th December (top right) as seen from the location of the local Automatic Weather Station. The Accumulation area of the Eastern Canin glacier is visible in the background of the picture. See people working on it in the right picture for scale. The bottom photograph (Renato R. Colucci) shows the outcropping glacier ice of Canin on 30th December, 2015. A Bergschrund and some small crevasses are visible.

Supplementary Table 1: Regional climate models used in the analysis of Figure 5. Also indicated are the global models driving the regional climate simulations (acronym before the slash sign) and whether the simulation was part of the EURO-CORDEX (EC) or MED-CORDEX (MC) programs. The regional model horizontal grid spacing for all simulations is 0.11°.

**Global/Regional Climate Model**	**Regional Modelling Group**
CNRM-CM5/ALADIN (MC)	Centre National de Recherches Meteorologiques, France
MPI-ESM-LR/CCLM (EC)	Climate Limited-area Modelling Community, Germany
CNRM-CM5/RCA4 (EC)	Swedish Meteorological and Hydrological Institute, Rossby Centre, Sweden
EC-EARTH/RCA4 (EC)	Swedish Meteorological and Hydrological Institute, Rossby Centre, Sweden
HADGEM2-ES/RCA4 (EC)	Swedish Meteorological and Hydrological Institute, Rossby Centre, Sweden
MPI-ESM-LR /RCA4 (EC)	Swedish Meteorological and Hydrological Institute, Rossby Centre, Sweden
IPSL-CM5A-MR /RCA4 (EC)	Swedish Meteorological and Hydrological Institute, Rossby Centre, Sweden
EC-EARTH /RACMO (EC)	Royal Netherlands Meteorological Institute, The Netherlands
MPI-ESM-LR /REMO (EC)	Max-Planck-Institut für Meteorologie, Germany
HadGEM2-ES /RegCM4 (MC)	International Centre for Theoretical Physics, Italy
